# Review of Developments in Combating COVID-19 by Vaccines, Inhibitors, Radiations, and Nonthermal Plasma

**DOI:** 10.3390/cimb44110384

**Published:** 2022-11-15

**Authors:** Ihn Han, Sohail Mumtaz, Sekar Ashokkumar, Dharmendra Kumar Yadav, Eun Ha Choi

**Affiliations:** 1 Department of Plasma Bio-Display, Kwangwoon University, Seoul 01897, Republic of Korea; 2 Plasma Bioscience Research Center (PBRC), Applied Plasma Medicine Center, Kwangwoon University, Seoul 01897, Republic of Korea; 3 Department of Electrical and Biological Physics, Kwangwoon University, Seoul 01897, Republic of Korea; 4 Gachon Institute of Pharmaceutical Science and Department of Pharmacy, College of Pharmacy, Gachon University, Hambakmoeiro 191, Yeonsu-gu, Incheon 21924, Republic of Korea

**Keywords:** nonthermal plasma, COVID19, SARS-CoV-2, NTAP viral inactivation, coronavirus disinfection

## Abstract

Global society has been highly pressured by the COVID-19 pandemic, which has exposed vulnerabilities in supply chains for disinfection products, personal protective equipment, and medical resources worldwide. It is critically necessary to find effective treatments and medications for these viral infections. This review summarizes and emphasizes critical features of recent breakthroughs in vaccines, inhibitors, radiations, and innovative nonthermal atmospheric plasma (NTAP) technologies to inactivate COVID-19. NTAP has emerged as an effective, efficient, and safe method of viral inactivation. NTAP can be used to inactivate viruses in an environmentally friendly manner, as well as activate animal and plant viruses in a variety of matrices. Researchers and engineers desire to help the medical world deal with the ongoing COVID-19 epidemic by establishing techniques that make use of widely available NTAP technologies. NTAP technology is not dependent on viral strain, and it does not necessitate months or years of research to develop specific vaccines for each novel or arising viral disease. We believe the NTAP is a highly promising technique for combating COVID-19 and other viruses. Thus, NTAP technology could be a significant breakthrough in the near future in assisting humans in combating COVID-19 infections. We hope that this review provides a platform for readers to examine the progress made in the fight against COVID-19 through the use of vaccines, inhibitors, radiation, and NTAP.

## 1. Introduction

COVID-19 is an epidemic infection created by SARS-CoV-2 (severe acute respiratory syndrome coronavirus-2), and it was initially spotted in Wuhan, Hubei province, China. This infection spread worldwide and was responsible for the last pandemic. In the past 72 years (specifically 1960, 1967, 2003, 2004, 2005, 2012, and 2019), seven types of viral infections have been identified. In the 1960s, the virus infected the respiratory tract and produced a common cold, but now these infections have created major impacts all over the world. In 1965, the human coronavirus (HCoV) B814 strain was isolated from the common cold, but this strain could not be propagated in tissue culture in a laboratory.

The HCoV-229E prototypic strains (named after a student specimen coded 229E) were isolated from human respiratory tract tissue infected with the common cold [1]. In 1967, HCoV-OC43 (organ culture 43) was isolated from a tracheal organ culture [2], and both strains induced the same clinical symptoms (fever, cough, nasal discharge, sneezing, sore throat, and shortness of breath). Severe acute respiratory syndrome coronavirus (SARS-CoV) induced atypical pneumonia in humans, and it primarily appeared in Foshan City, Guangdong province, China, in November 2002. HCoV was usually harmless until the spread of SARS-CoV. HCoV-NL63 (The Netherlands 63) was obtained from the aspirate of a 7-month-old infant with bronchiolitis in The Netherlands in 2004 [3], whereas human coronavirus-Hong Kong University 1 (HCoV-HKU1) was isolated from a 71-year-old man who had been infected with pneumonia in a Hong Kong hospital in 2005 [4]. After the SARS-CoV outbreak, the Middle East respiratory syndrome (MERS) produced a pandemic in 2012. This virus was isolated in the lung sputum samples of a 60-year-old man infected with fatal pneumonia and acute renal failure [5]. The severe acute respiratory disease COVID-19 was reported in the Hubei Province of China. This infection spread to other countries and promoted severe pneumonia in a worldwide pandemic [6]. The coronavirus generally causes mild to moderate upper-respiratory tract illness; additionally, it is associated with the common cold, fever, cough, and pneumonia. From epidemiological investigation, we know these viruses are spread worldwide in a seasonal pattern [7].

The emergence of new variants of viruses induces new types of infections; with these differences, particularly genetic variations in the spike protein, the infectivity, antigenicity, and/or vaccine efficacy of the pathogen are altered. The world health organization (WHO) classifies coronavirus variants into the following: variants of concern (VOCs), variants of interest (VOIs), variants being monitored (VBMs), and variants of high consequence (VOHCs) (see www.who.int). Previously virus VOCs included Alpha, Beta, Gamma, and Delta, and now include Omicron (B.1.1.529). The 2020 coronavirus variant Alpha was detected in the UK in September, Beta in South Africa in May, Gamma in Brazil in November, and Delta in India in October. Omicron genomic sequencing indicated that 60 different genetic mutations, compared with the original SARS-CoV-2, had a fast infection rate when compared with other variants and also had fewer effects.

### 1.1. Classifications

The tenth International Committee on Taxonomy of Viruses (ICTV) assessed virus taxonomy. Coronavirus (CoV) is categorized as follows: CoV belonging to Riboviria Realm, Orthornavirae Kingdom, Pisuviricota Phylum, Pisoniviriceses Class, Nidovirales Order, Cornidovirineae Suborder, and Orthocoronavirinae are phylogenetically subdivided into four categories: (i) αCoV (HCoV-229E, HCoV-NL63), (ii) βCoV (HCoV-OC43, HCoV-HKU1, MERS-CoV, SARS-CoV, SARS CoV-2), (iii) γCoV (Avian-CoV, Duck CoV-2714), (iv) δCoV (CoV-HKU15, White CoV-HKU16). αCoV and βCoV are found in mammals; γCoV and δCoV are found in birds. The origin, sources, and intermediate hosts are explained in Table S1. The human coronaviruses envelop positive-sense, single-strand-RNA viruses and genomes 29–32 kb larger than other RNA viruses. It is a natural origin of all the human coronavirus [8].

### 1.2. Virion Structure

SARS-CoV-2 has a single-stranded, positive-sense RNA genome with 29,891 nucleotides and 38% G + C content, encoding 9860 amino acids. SARS-CoV-2 has a variable number of open reading frames (ORF); these types of fragments are expressed as a nonstructural protein (3-chymotrypsin-like protease (3CLPro), papain-like protease (PLpro), RNA-dependent RNA polymerase (RdRp), and structural protein). The structural proteins are mainly the spike (S), membrane (M), envelope (E), and nucleocapsid (N) proteins. Between these, the ribonucleoprotein (N) capsid covers the E, M, and outer surface S [9,10], and protein characteristics are similar to other βHCoV. 

The HCoV-SARS-CoV-2 molecular characterization of the genomic structure was reviewed by [11,12]. The SARS-CoV-2 genome encoded two flanking untranslated regions (UTRs) and a single long open reading frame encoding a polyprotein, and the genome is organized in the order of 5′-replicase (ORF1/ab)-structural proteins (spike protein (S), envelope protein (E), membrane protein (M), nucleocapsid protein (N))-3′ and also the hemagglutinin-esterase (HE) gene, which is identified as βCoVs (HCoV-OC43 and HCoV-HKU1). Two-thirds of the viral genomic region (20 kb) and the head contains open reading frame 1a and ab (ORF1a, ab), which potentially encode the nonstructural proteins (NSPs) referred to as the pp1a (NSPs1 to NSPs11) and pp1ab (NSPs12 to NSPs16) polyproteins, and the remaining 10 kb region preceding 3′-end encodes various structural proteins (S, E, M, and N). In addition, the structural genes encode nine accessory proteins: the ORF3a, ORF3d, ORF6, ORF7a, ORF7b, ORF8, ORF9b, ORF14, and ORF10 genes [13].

### 1.3. Viral Inflammation

**Figure 1:**
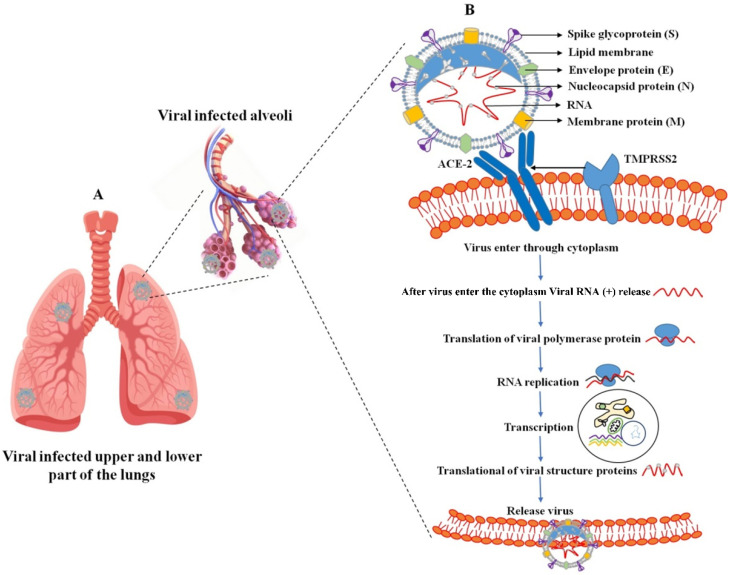
SARS-CoV-2 infection in the upper and lower part of the lungs (**A**); virus-infected host cell and virus propagation cycle (**B**). The SARS-CoV-2 virus consists of membrane proteins (M) in the lipid membrane along with envelope proteins (E) and spike glycoproteins linked to the envelope. RNA bound to the nucleocapsid protein exists inside the virus. External binding protein S can enter the lungs by binding to angiotensin-converting enzyme-2 (ACE-2) receptors present in human lung cells. In this case, the virus releases RNA together with genetic information and performs RNA replication to synthesize all components of the virus. Synthetic components are assembled and released out of lung cells.

Human coronaviral inflammation induces the clinical symptoms of fever, cough, and shortness of breath. These severe symptoms promote acute respiratory distress syndrome (ARDS), and ARDS promotes lung infection (Figure 1A). Several stages are involved in the life cycle of SARS-CoV-2. The virus enters the host cell through a cell membrane attachment, and fusions are mediated by the S glycoprotein, replication, transcription, assembly, and release. Two primary processes are involved in the virus entry: (I) receptor binding domain (RBD) and (II) proteolytic activity (transmembrane bound proteases serine 2 and cathepsin L). The SARS-CoV-2 surface contains an S protein that binds to the receptor [14], and it can be cleaved by a furin-like protease into two functional subunits, such as S1 and S2.

The receptor binding domain (RBD) of the S1 subunit contains the angiotensin-converting enzyme-2 (ACE2) responsible for binding to the virus receptor, and S2 mainly contains the HR domain. This involves virus fusion and is also divided into HR1 and HR2 [15]. Various types of protease involve the cleavage of proteins at the S1 or S2 sites on the host cell surface by the host transmembrane-bound protease serine 2 (TMPRSS2 early pathway) and utilize the endosomal cathepsin L (CatL) to enter using the late pathway [16,17]. These processes assess membrane fusion between the virus and host cell and also release the viral genome into the cytoplasm [17]. The virus enters the cytoplasm; the viral genomic RNA (sgRNA) is translated into the two polyproteins 1a and 1b (pp1a (ORF1a producing) and pp1b (ORF1b producing)). These proteolytic activities assess the 15–16 nonstructural proteins (NSPs), and each protein has its own specific function. Among them, NSP12 (RNA-dependent RNA polymerase (RdRp)) assembles with several NSPs to form a viral genome replication and transcription complex (RTC). The NSPs 3, 4, and 6 prompted cellular membranes to form double-membrane vesicles (DMVs) [17,18,19]. Transcription of a 5′-set of nested negative-sense sub-genomic RNA molecules (sgRNAs) is used to synthesize the 3′-set of nested positive-sense sgRNAs [20,21]. The structural proteins (S, HE, M, and E) and other related accessory proteins are transformed into the endoplasmic reticulum (ER), but the N protein is translated by cytosolic ribosomes. The assembled virus particles take place in the ER-Golgi intermediate compartment (ERGIC), and new virions are released through exocytosis (Figure 1B). The main functions of a structural protein are S protein (virus entry and fusion), M and E proteins (assembly and production of the virion), and N protein (binds with viral genome and virus release).

**Figure 2:**
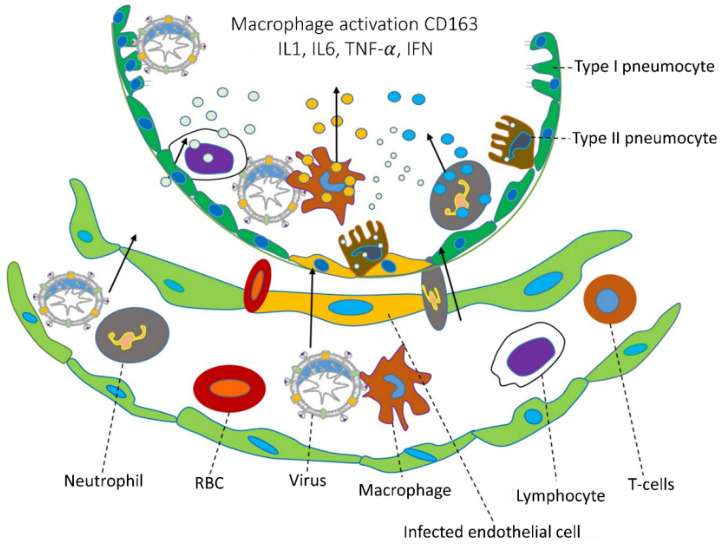
Coronavirus infections and immune approach. During coronavirus infections, the lower part of the lungs collects fluid in the bronchioles and disrupts surface coating types I and II pneumocytes. Additionally, they disrupt the immune response and increase cytokine release, which leads to the accumulation of reactive oxygen species. Various cytokines are secreted by CD163 macrophages activated by a viral infection in infected endothelial cells, and immune responses are caused by IL1, IL6, TNF-α, IFN, etc. Chemical catalysts by this cytokine attract various immune cells, such as neutrophils, lymphocytes, and T cells, through blood vessels. (The specific cells are indicated by dotted lines, and the direction of cell movement is indicated by an arrow).

At this point, there are no specific antiviral drugs, and before developing the vaccine, we must know how to infect the host cells and what is happening inside the cell. Coronavirus infection promotes lung damage, and those inflammations increase the cytokines and chemokines produced in vivo leading to infection of the alveolar epithelial cells and capillary endothelial cell damage (Figure 2) [22]. After viruses enter a cell, the adaptive immune system responds against an antigen, and B and T cells are involved in this process. The infection cytokines produced by interleukin (IL-6, IL-8, IL-1β), tumor necrosis factor-alpha (TNF-α), and interferon-gamma (IFN-γ) are all accompanied by a severe infection [23]. The chemokines produced were chemokine ligand 3 or macrophage inflammatory protein 1 alpha and 1 beta (MIP1α and 1β), C-C motif chemokine ligand 2 (CCL2), chemokine ligand 5 and 20 (CCL5, CCL20), C-X-C motif chemokine ligand (CXCL1, CXCL2, CXCL8, CXCL10, and CXCL17) [24]. Interleukin-6 (IL-6) is responsible for the innate and acquired immune response and also several other activities during the improvement of acquired immunity against infections, while also responsible for antibody fabrication of B cells and differentiation of dendritic cell and T cell regulations. TNF-α is responsible for cytokines for hyperinflammation through viral infections and cytokines secreted by macrophages, Th17 cells, CD8^+^ T cells, and DCs. C-reactive protein (CRP) is the prototype of human acute phase proteins (APPs). CRP plays an important role in innate immunity as an early defense mechanism against infections. The elevated levels of IL-6, TNF-α, and C-reaction protein (CRP) have caused more lungs damages. There is currently no treatment for long-term symptoms caused by a SARS-CoV-2 infection. This could be the next major issue to address. Veronese et al. (2022) found olfactory dysfunction from long COVID-19; they used sniff sticks to reduce COVID-19 symptoms, which were excluded by olfactory training/stimulation for up to 30 days compared with the control [25].

## 2. FDA-Approved and Emergency-Approved Drugs against SARS-CoV-2

The United States Food and Drug Administration (FDA) and the European Medicines Agency (EMA) have approved remdesivir drugs against SARS-CoV-2, and other drugs are unauthorized. Remdesivir (Veklury) intravenous (IV) injection, made by Gilead Sciences under the brand Veklury, was originally used against Ebola and hepatitis C and is now used against SARS-CoV-2; the FDA approved this in October 2020. Produced by Pfizer, Paxlovid (PF-07321332) combined nirmatrelvir and ritonavir oral drugs and was previously used for a SARS-CoV infection; from December 2021 onwards, it was used against SARS-CoV-2. Molnupiravir (Lagevrio), orally administered (two times per day up to 5 days), was developed by Ridgeback Biotherapeutics and Merck. In December 2021, the FDA accepted this drug to fight influenza, and in January 2022, India announced molnupiravir drugs were used for SARS-CoV-2 infected patients. Sotrovimab and bebtelovimab (175 mg/2 mL) enhance the monoclonal antibodies (mAbs) and inhibit the spike protein, and GlaxoSmithKline and Vir Biotechnology, Inc developed IV injections for both, and they were approved in May 2021 and February 2022, respectively. Tocilizumab (Actemra) is an IV injection that blocks the inflammation of protein IL6 and it was accepted in June 2021. Baricitinib (Olumiant) is orally taken as one tablet daily for up to 14 days and was accepted in November 2020. Bamlanivimab and etesevimab enhance monoclonal antibodies (mAbs) and are recommended for use against the Omicron variant; they were accepted in January 2022. REGEN-COV (casirivimab and imdevimab) are monoclonal antibodies (mAbs) that target the viral spike protein to prevent it from entering and infecting healthy cells; this one was approved in November 2020. 

The EU developed and adapted vaccines against different SARS-CoV-2 variants. The intramuscular (IM) injected Evusheld, a combination of Tixagevimab and Cilgavimab (formerly AZD7442), treated COVID-19 (TACKLE Phase III). The Food and Drug Administration, on March 28, 2000, accepted the emergency use of the monoclonal antibody sotrovimab to treat against omicron BA2 subvariant of coronavirus.

## 3. Vaccine

At this time, the FDA and EMA have approved three vaccines against SARS-CoV-2 (Moderna, Pfizer-BioNTech, and Johnson & Johnson’s Janssen), and also the one-off is now approved by EMA (AstraZeneca). The vaccine protects against human coronavirus infection and has been developed by other companies with other aspects, particularly cell culture-based vaccines (European medicines agency Flucelvax Tetra) (Bühler and Ramharter, 2019), vector vaccines, DNA and RNA vaccines, and recombinant protein vaccines against hepatitis B virus [26,27]. Spike proteins are a primary target for developing the vaccine against SARS-CoV and SARS-CoV-2. The homotrimeric S protein involves viral host and tissue tropism and RBD protein-inhibited SARS-CoV-2 entry into angiotensin-converting enzyme (hACE2) [28]. The SARS-CoV S-protein contains two subunits (S1 and S2); the S1 subunit has four domains, of which the receptor binding domain (RBD) is central as it binds to the ACE-2, and S2 is involved in viral fusion into the host cell membrane. The S protein enhances protective immunity with the initiation of neutralizing antibody and T cell responses [29,30,31]. There is an urgent need for an effective SARS-CoV-2 vaccine; this includes genetic-based (mRNA and DNA) [32,33], replicating and non-replicating viral vectors (measles, adenovirus, and baculovirus) [34], recombinant proteins or peptides, virus-like particles (VLPs) and nanoparticles, or inactivated and live-attenuated viral vaccines [35,36,37,38]. The World Health Organization (WHO) reported many pharmacological companies and researchers developed SARS-CoV-2 vaccines; 199 vaccines are in preclinical trials, and 172 vaccines are clinically developed out of phase 1 (53), phase 1/2 (4), phase 2 (3), phase 2/3 (3) and phase 3 (6) (WHO, 1 November 2022, (Table 1)).

### 3.1. Protein Subunit Vaccines

This approach has been used in the development of the majority of SARS-CoV-2 vaccine candidates in both the clinical and preclinical stages. Only essential viral proteins or peptides that can be produced in vitro in bacteria, yeast, insect, or mammalian cells are used in this technique. For the development of a vaccine against viral infections, the spike protein is a key step in developing the vaccine because, during viral infection, the spike protein is first attached to the host cell. SARS-CoV-2 containing spike protein enters the host cells via angiotensin-converting enzyme 2 (ACE2) [39]. The RBD binding the S protein induces the neutralizing antibodies (NAbs) and T cell immune responses and also inhibits virus entry [40]. The vaccine contains the whole length of the SARS-CoV-2 S protein; its main goal is encroaching to neutralize the antibody and similar level of efficacy in SARS and MERS vaccines [41,42].

### 3.2. Viral-Vector-Based Vaccines

Pathogen antigen-encoding genes are cloned into replicating or non-replicating virus vectors (such as adenovirus). After immunization, transduced host cells generate the antigen(s). Recombinant viral vectors are used as a joint platform to express the transgene and produce immunogenic antigen [43], and the vector is expressed once into the infected human cells, eliciting the antigen-specific humoral and cell-mediated immune responses via antigen presentation [44]. Manufacturing effective viral vector vaccines is a complicated process, such as cellular optimization and preventing contamination [45]. Different types of vectors are used, such as MERS-CoV (human adenovirus type 5 (Ad5) and type 41 (Ad41)) [46,47].

### 3.3. Nucleic Acid-Based Vaccine

The advantage of nucleic acid (DNA and RNA) vaccines against emerging infections is that they are quick to manufacture. In the case of DNA vaccines, host cells use a sequential transcription-to-translation process to create the viral antigen(s) encoded by a recombinant DNA plasmid. In contrast, mRNA vaccines are created via in vitro transcription and produce viral antigen(s) in the cytoplasm through direct protein translation in vivo. The produced nucleic acid-based vaccines are interesting for the development of new antibodies and easy-to-develop vaccines. Delivering the vaccines for in situ methods and sending the genetic codes for the in situ production of viral proteins is a promising alternative to conventional vaccine approaches. The nucleic acid-based vaccine promotes the innate and antigen (adaptive) immune response [48]. On 23 February 2022, the WHO clinically recognized RNA and DNA vaccines, as stated in Table S2 in the supplemental information. The RNA vaccine established from the messenger RNA (mRNA) enters into the host cell and encroaches on humoral and cellular immunity [49]. Developing RNA-based therapies mainly involves replicating viral proteins into the host cell. We reviewed the RNA-based strategies and their prospects for COVID-19 treatment [50]. Several factors are involved efficacy of mRNA vaccines, such as ensuring mRNA purity and others, particularly adding 5′ Kozak and cap sequences and 3′ poly-A sequences. Modified nucleosides increase mRNA stability and decrease detection by the receptors of innate immune cells, codon optimization, and also intradermal injection to reduce RNA degradation by generating thermostable mRNA [51,52]. DNA-based vaccines are unclear and less potent compared with RNA vaccines. DNA-based vaccines consist of a fragment of genetic code encoding antigens into the host cell using DNA plasmids as a vector; this attitude enhances the cell-mediated and humoral immune responses [53].

### 3.4. Live-Attenuated Virus Vaccines

In this method, a virus is attenuated through reverse genetic mutagenesis, in vitro or in vivo passage. By simulating a live virus infection, the resultant virus loses its ability to cause disease or becomes only mildly infectious. Inactivated and live-attenuated viruses use the whole virus as their target. Live-attenuated virus vaccines can also produce nonstructural and accessory proteins in vivo. They contain all structural proteins (S, N, M, and E proteins), which are based on a single protein or protein fragment. These vaccine candidates can elicit a wider range of antibody and T cell responses. One vaccine candidate is in phase I/II clinical studies, while three vaccine candidates based on an inactivated virus approach are currently in phase III clinical trials. In preliminary research, mice were given the inactivated viral vaccination PiCoVacc, and it developed antibodies against the S protein (including RBD-specific antibodies) and the N protein. PiCoVacc and Sinopharm COVID-19 Vaccine BBIBP-CorV, another inactivated viral vaccination, significantly increased the generation of nAb in NHPs but did not trigger T cell responses. IBV, TGEV, BCoV, and FIPV140 live-attenuated viral vaccines have been approved for use in animals. Further research is required to determine how CoV proteins in the live virus relate to immunological memory impairment. These vaccinations typically require a lengthy process of viral attenuation. Only three live COVID-19 vaccines with codon-deoptimization as their attenuation method are currently being tested in humans.

## 4. Inhibitors

### 4.1. SARS-CoV-2 3CLpro Protease Inhibitors

The primary betacoronavirus protease, 3-chymotrypsin-like protease (3CL^pro^), also known as Mpro, is required for viral RNA translation in polyprotein synthesis [54,55]. SARS-CoV-2 3CLpro x-ray structures and ketoamide complexes have recently been reported [54]. Within 4 to 24 h of dosing, two compound pulmonary tropism pyridine-containing ketoamides, 13a and 13b, exerted pharmacokinetic properties in the lungs and bronchial-alveolar liquid lavage of mice.

**Figure 3:**
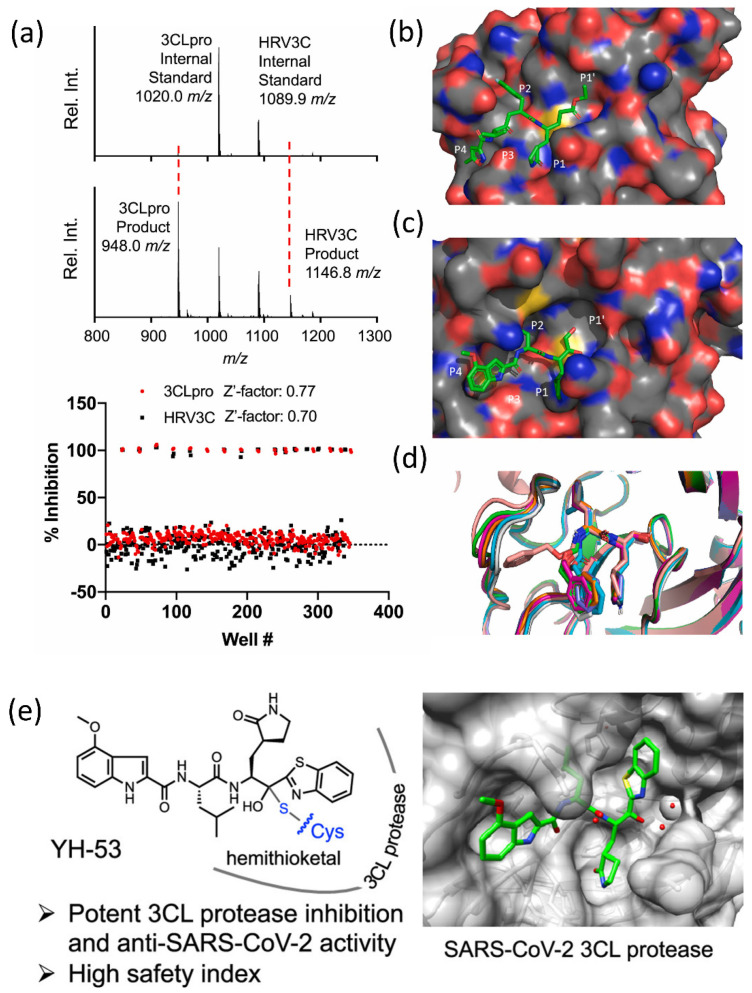
(**a**) SAMDI-MS spectra of duplexed reactions before and after 3CLpro and HRV3C activity (top and bottom). (**b**–**d**) Structural analysis of protease inhibitor binding sites. Rupintrivir binding mode in HRV3C protease (**b**,**c**). (**d**) Superimposition of previously reported SARS-CoV-2 3CL-pro structures with bound GC376. Reprinted with permission from [60]. Copyright 2021, Elsevier. (**e**) The most powerful and effective inhibitor, YH-53, can effectively prevent SARS-CoV-2 replication. Reprinted with permission from [59]. Copyright 2022, American Chemical Society.

**Figure 4:**
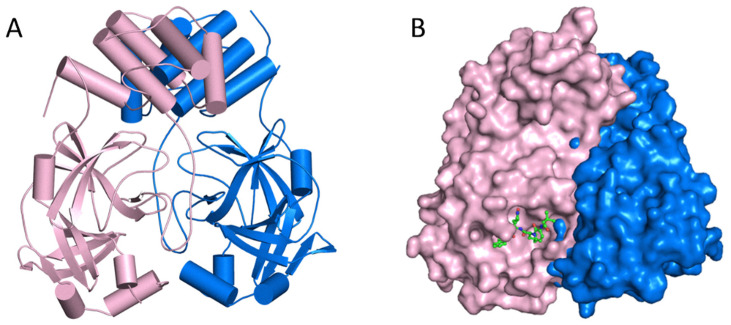
(**A**) The major protease of the SARS-CoV-2 dimer’s structural characteristics. The primary protease of the SARS-CoV-2 virus. (**B**) In the active site groove, a sphere represents the primary protease monomer.

The SAMDI-MS spectra of duplexed reactions before and after 3CLpro and HRV3C activity are shown in Figure 3a (top and bottom). The structural analysis of the binding sites for protease inhibitors is shown in Figure 3b–d. Figure 3b,c show the HRV3C protease’s mode of rupintrivir binding. The superimposition of previously published SARS-CoV-2 3CL-pro structures with bound GC376 is shown in Figure 3d. N3, formerly known as the Michael acceptor inhibitor, was created with a computer-aided method. N3 has shown significant antiviral efficacy against infectious bronchitis viruses in an animal model and can selectively inhibit the Mopar of numerous coronaviruses, including SARS-CoV and MERS-CoV [56,57,58]. SARS-CoV-2 3CLpro was demonstrated to establish a covalent link with N3, acting as an irreversible inhibitor of SARS-CoV and MERS-CoV 3CLpro. Recently, peptidomimetic compounds with a unique benzothiazolyl ketone as a warhead group were reported to have potent anti-SARS-CoV-2 3CLpro activity (Figure 3e) [59]. The most potent inhibitor, YH-53, can effectively prevent SARS-CoV-2 replication. According to X-ray structural analysis, YH-53 forms multiple hydrogen bonds with backbone amino acids and a covalent bond with the active site of 3CL^pro^. The structural features of the SARS-CoV-2 dimer are depicted in Figure 4A. the virus SARS-main CoV-2’s protease. Figure 4B shows that the primary protease monomer is represented by a sphere in the active site groove.

### 4.2. Mpro Inhibitors

**Figure 5:**
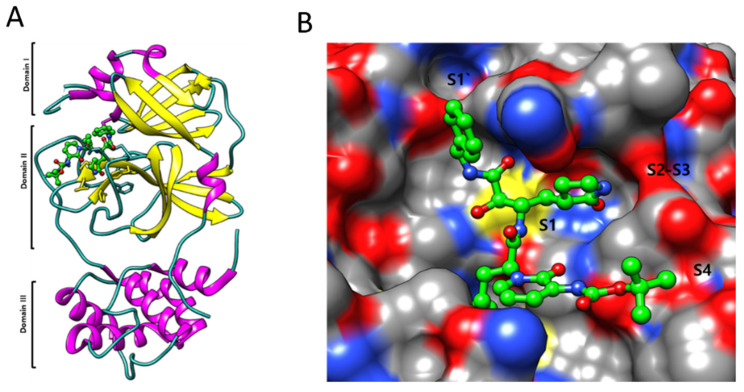
(**A**) The major protease of the SARS-CoV-2 monomer has three domains and is structurally unique. A linker connects Domains II and III, which is necessary for protein dimerization. (**B**) Subsite groups in the substrate-binding subsites of SARS-CoV-2 MPro: S1′, S1, S2, S3, and S4.

The apo form of SARS-CoV-2 Mpro and ketoamide coupled to the protein form a dimer crystallographic structure composed of two monomers with identical conformations. Each protomer is composed of three domains, as illustrated in Figure 5A,B, with Domains I (residues 8–101) and II (residues 102–184) interacting to produce the protein’s active site, which is constructed of a Cys145-His41 dyad with an β-parallel-barrel structure. Domain III (residues 201–303) has five helices and is related to Domain II by a lengthy loop region (residues 185–200), with the substrate-binding site in a cleft between Domains I and II. It establishes a catalytic connection with the His41 and Cys145 and neighboring residues in the substrate binding cleft, such as the Gly143 and Ser144 dyad, and has multiple highly conserved interactions. SARS-CoV processes and cleaves its lengthy polyprotein precursor into individually functional nonstructural proteins using a chymotrypsin-like protease (3CLpro) and a papain-like protease (PLpro) [61,62].

The S1 residues provided by Cys145, Gly143, and Ser144 also function as the oxyanion hole in the active site area of SARS-CoV-2 Mpro. The S4 site is formed by bulky Gln189 and Pro168 residues, the S1 residue is His163, and the S2 position is occupied by Glu166 and Gln189 (Figure 5A). To decide the commencement of proteolysis and the creation of nonstructural proteins for the development of the replication-transcription complex, the Mpro identifies and binds particular residues at each subsite of the peptide substrate. The most variable regions of Mpro are the helical Domain III and surface loops, while the substrate-binding pocket (located in a cleft between Domains I and II) is highly conserved across all coronaviruses, as shown in Figure 5B. This suggests that antiviral inhibitors targeting this pocket should have broad antiviral activity against coronaviruses. Mpro possesses a substrate-recognition pocket that is highly conserved across all coronaviruses, according to a prior study, and this pocket could be used as a therapeutic target for the development of broad-spectrum inhibitors. The recent discovery of novel coronaviruses, as well as the gathering of structural data for Mpro from coronaviruses of various species, has allowed this concept to be investigated further.

## 5. Virus Deactivation by Radiation

Viruses are the world’s most abundant and varied microorganisms. They’ve been on the planet for billions of years [63], have evolved to a variety of environments, and can now be found in all ecosystems. Viruses have played a role in the evolution of life on Earth, and they can help to preserve ecosystems and essential natural Earth cycles, such as the carbon cycle in the ocean [64]. Pathogenic viruses, on the other hand, cause tens to hundreds of millions of plants, animal, and human infections each year, resulting in significant agricultural losses and countless fatalities. To improve quality of life, it is necessary to inactivate dangerous viruses. Radiations are well known to have several effects when interacting with biological systems [65].

### 5.1. Ultraviolet Radiations

**Figure 6:**
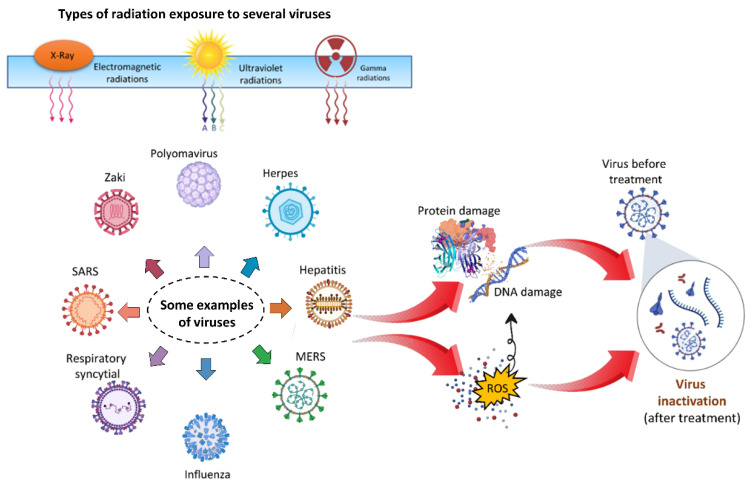
Illustration of the direct and indirect impacts of several types of radiation on microorganisms. Radiation can cause direct DNA and protein damage which leads to virus inactivation. On the other hand, radiation can also cause the generation of ROS in response to oxidative stress, which also causes to damage the DNA and proteins of microorganisms and lead to inactivation.

For the decontamination of various virus species, a variety of physical approaches have been used, including electromagnetic irradiation, X-rays, UV, and gamma radiations [66,67,68,69,70,71,72,73]. Many radiation sources exist, and radiation production is an ongoing subject of study [74,75,76,77,78,79,80,81,82,83,84] that is beneficial to facilitate our modern lives. Figure 6 graphically depicts the direct impact and mechanism of employing radiations to disinfect microorganisms. Irradiation is a low-energy, ecologically friendly, and safe way of killing viruses under carefully regulated conditions with few molecular modifications, which is particularly significant in the manufacture of biological reagents. The linearity of the fatal dosage effects and the ability to measure the dose to be provided are two major benefits of irradiation over other approaches [85].

The sun’s UV radiation in the atmosphere is the most effective natural germicide. Far-UVC light with a wavelength range of 207–222 nm, on the other hand, probably eliminates germs without harming their natural equivalents [86]. UV absorption and viral inactivation are both aided by nucleic acids found within pathogens. UV radiation, with a wavelength of 254 nm, inactivates and tabulates the susceptibilities of a wide variety of viruses, including those with double- and single-stranded RNA or double-stranded DNA genomes [87,88]. With solar inactivating bio-threat viruses, two issues must be considered: 1) determining the UV sensitivity of various viruses and 2) knowing the number of RNA or DNA bases to determine the sensitivity to UV inactivation at a specific UV wavelength [89]. Furthermore, pyrimidine dimers, particularly thymine dimers, which are the most naturally toxic UV photoproducts, cause significant variations in sensitivity between many viral replication types. DNA viruses are more vulnerable to UV damage than RNA viruses because DNA contains thymine [90]. SARS-CoV2 can be successfully inactivated by UVC irradiation, according to a recent study [88,91,92,93,94,95]. Recent research has also emphasized the use of UV radiation to decontaminate N95 respirators to assure COVID-19 safety [96].

### 5.2. Gamma Radiations

Gamma radiation is ionizing radiation with the smallest wavelength, and most of the energy in the electromagnetic spectrum is capable of inactivating DNA and RNA viruses [97,98,99]. The breakdown of DNA and RNA by radiolysis or genetic material cross-linking is assumed to be the main process behind the inactivation of viruses by irradiation [100]. In other words, it can either directly break down the DNA helix or produce free radicals that cause DNA damage [101]. The use of gamma irradiation to sterilize harmful organisms in the environment is efficient. Gamma radiation is commonly used in the sterilization of medical devices, injectable goods, and food samples due to its great decontamination capabilities [102]. Numerous studies have shown that gamma radiation is effective at inactivating pathogenic viruses in laboratory settings [98,103]. Gamma radiation sterilization has been used to study polyoma virus [104], vaccinia [105], influenza [106], and hepatitis A viruses [107].

Because of the present global COVID-19 epidemic, numerous researchers have looked at how effective gamma radiation is at inactivating the virus. Gamma radiation has been claimed to play a key role in vaccine manufacturing via viral inactivation since it has previously demonstrated efficacy in inactivating other enveloped viruses [108,109]. It was recently discovered that the volume and protein content of a sample influenced the viral inactivation performed by gamma irradiation using a surrogate virus [98]. X-rays can stop the virus from regressing by stopping cellular division and causing pathological alterations that eventually kill the virus [110]. Ionizing radiation has proven to be a highly successful approach to disinfecting gloves, surgical masks, and other items in the case of SARS-CoV-2 [111].

### 5.3. X-rays

Food disinfection and sterilization using X-ray irradiation is a well-known method. A lot of energy is released when an electron ray is used on a metal foil [112]. In comparison with X-rays, irradiation, thermal, or chemical decontamination methods are ineffective. It is a type of ionizing radiation that emits highly penetrative photons and has been shown to effectively inactivate viral species from several families [113]. A +10 voltage in an X-ray machine using a Machlett OEG-60 tube has been shown to reduce influenza virus survival [114,115]. Virus-induced papillomas in rabbits were found to be regressed after X-ray irradiation. The study further investigated if X-rays can slow virus regression by interfering with cellular division and causing pathological changes that eventually kill the virus [110]. For SARS-CoV2, ionizing radiation has proven to be an extremely effective method of sterilizing medical and other equipment [111].

## 6. Virus Deactivation by Nonthermal Atmospheric Plasma (NTAP)

### 6.1. The Introduction of NTAP

American scientist Irving Langmuir initially characterized plasma as one of the four fundamental states of matter in 1922 [116]. Plasmas are gases that have been totally or partially ionized. To remove electrons from atoms, enough energy is given to the gas, resulting in a combination of free electrons, free radicals, neutrals, and positively charged species [117,118,119]. Plasma is the most common type of matter, composing 99% of the total mass of the universe. Plasma can be found in the sun and other stars, galaxies, etc. Plasma can be m artificially ade with thermal and nonthermal properties.

There are two types of plasma: thermal plasma and nonthermal plasma. In a thermal plasma, all the particles are roughly the same temperature and are nearly completely ionized. Thermal plasma was also known as hot plasma and equilibrium plasma. Depending on the requirements, equilibrium plasma is used in a variety of applications [120,121,122,123,124]. Nonthermal plasma, on the other hand, has significantly lower temperatures than light electrons because heavy atoms and molecules are frequently near room temperature. The NTAP was also known as cold plasma and non-equilibrium plasma. Because NTAP is applied at room temperature, it is suitable for treating a wide range of biological materials, including solids, liquids, and aerosols. There are numerous applications for the two types of NTP, low pressure and atmospheric pressure [116,124,125,126,127,128,129,130]. Plasma medicine is a developing field that is attracting researchers from various disciplines, such as engineering, plasma physics, biology, and medicine [131]. When compared with other vaccines and radiations, NTAP is considered the most promising therapy against cancers and viruses because it has no harmful effects on healthy organs in biological systems [132].

**Figure 7:**
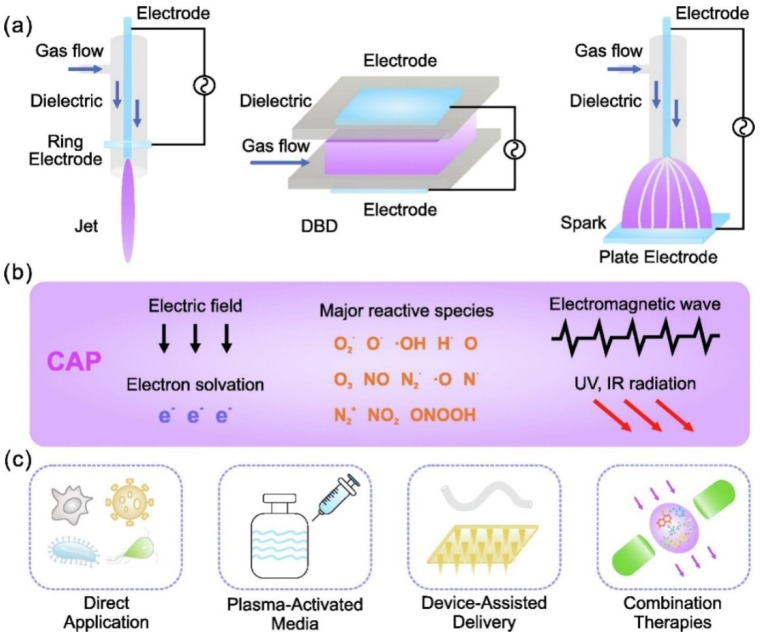
(**a**) Typical cold atmospheric plasma configurations for biomedical applications, such as plasma jet, dielectric barrier discharges (DBD), and spark; (**b**) plasma environment, which includes plasma generating reactive species, electrons, other ions, emissions, waves, and physical forces; (**c**) plasma delivery strategies, which include direct application, plasma-activated media, device-assisted delivery, and a combination of these. Reproduced with permission from [116]. Copyright 2022, Elsevier.

As shown in Figure 7, NTAP is composed of free electrons, atoms, and molecules in neutral, ionized, and/or excited states, including reactive oxygen species (ROS) and reactive nitrogen species (RNS) [133]. Thermal plasmas are usually completely ionized plasmas with roughly the same temperature for all particles [120]. Light electrons, on the other hand, have much higher temperatures in NTAP than heavy atoms and molecules, which are frequently comparable to room temperature. NTAP is suitable for treating a wide range of biological materials, including solids, liquids, and aerosols, because it is room temperature at the application site [124,134,135,136,137,138,139].

### 6.2. ROS Production by NTAP and Their Importance in Virus Inactivation

ROS are valuable cell-signaling molecules that are frequently produced in response to both extrinsic and intrinsic stimuli. ROS production, on the other hand, can damage a range of cellular components and events, distorting the normal function of the biological system [140]. When assessing a cell’s peroxidation, keep in mind whether an increased oxidant situation causes biomolecule damage and identifies the threshold for cell activities mostly through redox signaling [141]. NTAP generates a broad array of reactive species based on various variables, such as working gas, nature of the target, energy, and spacing between NTAP and target sample [126,142,143]. NTAP-generated free excited electrons with sufficient energy can electrically excite, dissociate, and ionize molecules, resulting in over 80 different species in the air [144,145]. ROS produced by plasma was required for pathogen deactivation. As a result, NTAP is emerging as a possible future method of virus deactivation [128].

### 6.3. Applications and Mechanism of NTAP for Virus Deactivation

**Figure 8:**
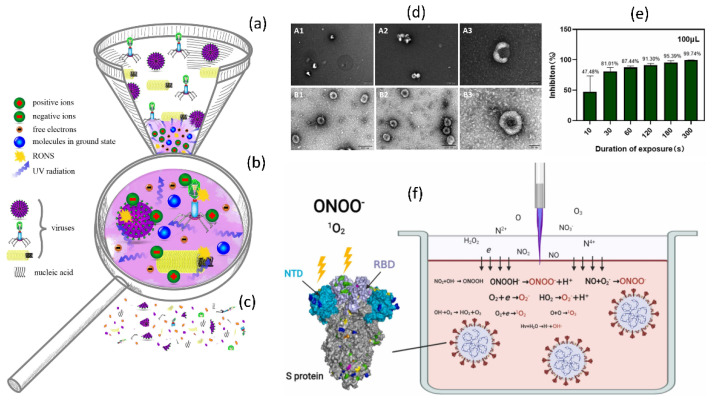
The mechanism of viral inactivation by plasma. (**a**) Morphological differences between viruses exposed to plasma. (**b**) A magnified view of the plasma properties responsible for viral inactivation. (**c**) These virus particles are partially or completely destroyed after being exposed to plasma, resulting in non-infective components. Reprinted with permission from [127]. Copyright 2020, Elsevier. (**d**) TEM images of GX_P2V before and after 3 min of CAP exposure. (**e**) Disinfection of virus with plasma exposure. (**f**) The mechanism through which RONS is produced in Ar-based NTAP damaged S protein. Plasma jet-generated reactive species dissolve into liquid and cross-react in the liquid phase. RONS ONOO^−^ and O_2_^−^ oxidized tyrosine, tryptophan, and histidine are predominate at RBD and NTD, thereby impairing RBD’s capacity to bind to cell receptor ACE2 and NTD’s function. After 3 min of cold atmospheric plasma (CAP) or NTAP treatment, the viral genome remained intact. Reprinted with permission from [146]. Copyright 2022, Elsevier.

In addition to direct plasma discharge to target samples, plasma-activated medium (PAM) or plasma-activated water (PAW) can be formed by exposing a biological liquid to electrical discharge under ambient conditions. A specific aim for plasma-related biological applications may need off- or on-site exposure circumstances. The active plasma plume comes into direct contact with biological items in on-site conditioned treatments because it is quite near to the ambient environment. In the off-site approach, plasma is immediately exposed to biological solutions (supplemented with a variety of RONS) for specialized biological applications. Surprisingly, these plasma-exposed treatments may be used in situations when plasma formation is not an option. They are also known as plasma-activated solutions (PAS) and can be used as therapeutic medicines. Changes in the feeding gases, flow rates, and given voltages can all affect the amount of RONS formed. Long-lived species can be stored and used later, whereas short-lived species can be used immediately. Plasma technology is simple and low-cost, making it suitable for a wide variety of applications. Depending on the treatment method, circumstances, and plasma supply, the viral inactivation mechanisms could be ROS, RNS, or a combination of both as shown in Figure 8a–c. As shown in Figure 8d–f, NTAP exposure has recently been shown to have inhibitory effects on SARS-CoV-2-like coronaviruses.

**Figure 9:**
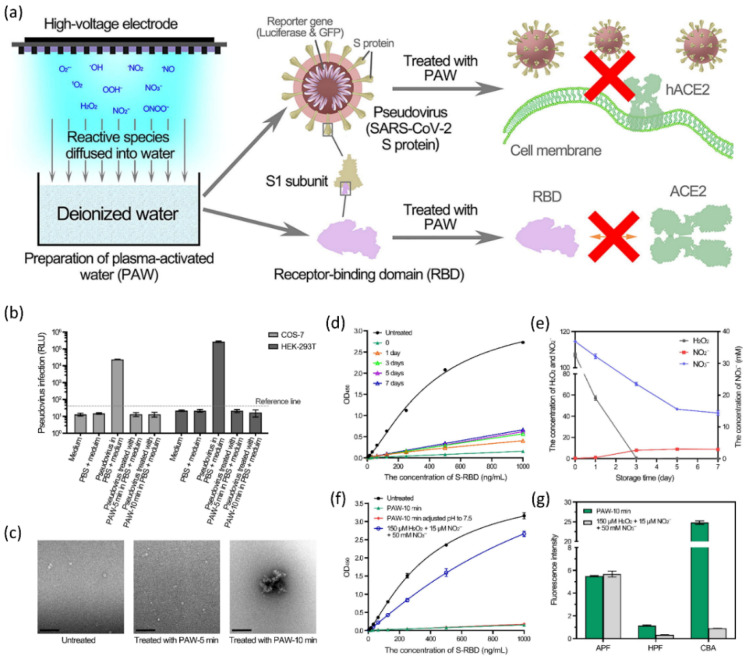
(**a**) A graphical representation of NTAP water treatment to prepare PAW and its application to COVID-19. (**b**) PAW inactivation of pseudovirus. Infection of COS-7 and HEK-293T cells with control and PAW-treated pseudovirus. (**c**) TEM analysis of PAW-treated pseudoviruses. The pseudoviruses treated with PAW-5 min and PAW-10 min, as well as the untreated pseudovirus, were negative stained and TEM examined. (**d**) The effects of PAW inactivation after storage. (**e**) Storage of long-lived species. The concentrations of H_2_O_2_ and NO_2_/NO_3_ in PAW-10 min stored for the indicated times were measured. (**f**) A comparison of the inactivation effects of PAW and a mixture of H_2_O_2_, NO_2_, and NO_3_. (**g**) Short-lived species in PAW and the H_2_O_2_, NO_2_, and NO_3_ mixed solution. Reused with permission from [147]. Copyright 2021, Elsevier.

Figure 9 shows the results obtained by using PAW for the inactivation of a pseudovirus, which is recently reported [114]. Figure 9a indicates the schematic of the NTAP device to prepare PAW by injecting reactive species into the water in the reported study. This prepared PAW was later applied to the virus for analysis. Figure 9b depicts untreated and PAW-treated pseudovirus inactivation and infection on COS-7 and HEK-293T cells. Pseudoviruses treated with PAW-5 min and PAW-10 min, as well as untreated PBS pseudoviruses, were mixed with four volumes of the medium. For infection, the mixture was introduced to COS-7 and HEK-293T cells. Figure 9c depicts the transmission electron microscopy (TEM) evaluation of PAW-treated pseudoviruses. The pseudoviruses treated with PAW-5 min and PAW-10 min, as well as the untreated pseudovirus, were negative stained and TEM examined. Figure 9d shows the effects of PAW inactivation after storage. PAW-10 min was stored for the specified periods, and the effects of inactivation on the COVID-19 RBD were investigated. Figure 9e depicts the storage of long-lived reactive species inside PAW. The concentrations of H_2_O_2_ and NO_2_/NO_3_ in PAW-10 min stored for the indicated times were evaluated in the reported study [114]. Figure 9f depicts a comparison of the inactivation effects of PAW and a mixture of H_2_O_2_, NO_2_, and NO_3_. The COVID-19 RBD was successfully inactivated by PAW-10 min, PAW-10 min with a pH maintained at 7.5, and a mixed solution of H_2_O_2_ (150 M), NO_2_ (15 μM), and NO_3_ (50 mM). Figure 9g depicts short-lived species in PAW and the H_2_O_2_, NO_2_, and NO_3_ mixed solution.

Furthermore, in contrast with other disinfection methods, NTAP is sometimes used as a novel, effective, cost-free, safe, and environmentally friendly substitute for inactivating viruses during decontamination procedures [112,115]. The NTAP strategy, like many others, could be improved. NTAP has a significant impact on water pH and conductivity because they are closely related to ion generation in the aqueous phase. When NTAP was applied to the water, nitrite species formed, lowering the pH and increasing conductivity due to ionic species [116]. When it came to the inactivation of viruses inside water, salinity and temperature also had a substantial role [117]. Salinity and temperature are associated because salinity has a greater impact at lower temperatures. According to research, temperature has a more significant influence than salinity alone in reducing viral persistence in water [118]. Viral persistence at low temperatures can be increased by high salt. However, minute temperature changes have nonlinear effects on the persistence of viruses and less NTAP inactivation efficiency.

Furthermore, the working gas in NTAP technology and device types are also important to achieve better inactivation inside water. It is well documented that the RONS generation and concertation are highly dependable on the NTAP device types, working gas, flow rate, and distance from the device to water [119]. The RONS generated by NTAP successfully inactivated the SARS-CoV-2 inside the water. This process is how the S protein is harmed by RONS generated in Ar-based NTAP. Reactive species produced by plasma jets dissolve into liquid and interact with one another inside. Tyrosine, tryptophan, and histidine were oxidized at RBD and NTD by the predominate RONS ONOO- and O2, which affected RBD’s ability to bind to the ACE2 cell receptor and NTD’s functionality. The viral genome remained unaltered after 3 min of treatment with NTAP [113], as shown in Figure 8f.

**Figure 10:**
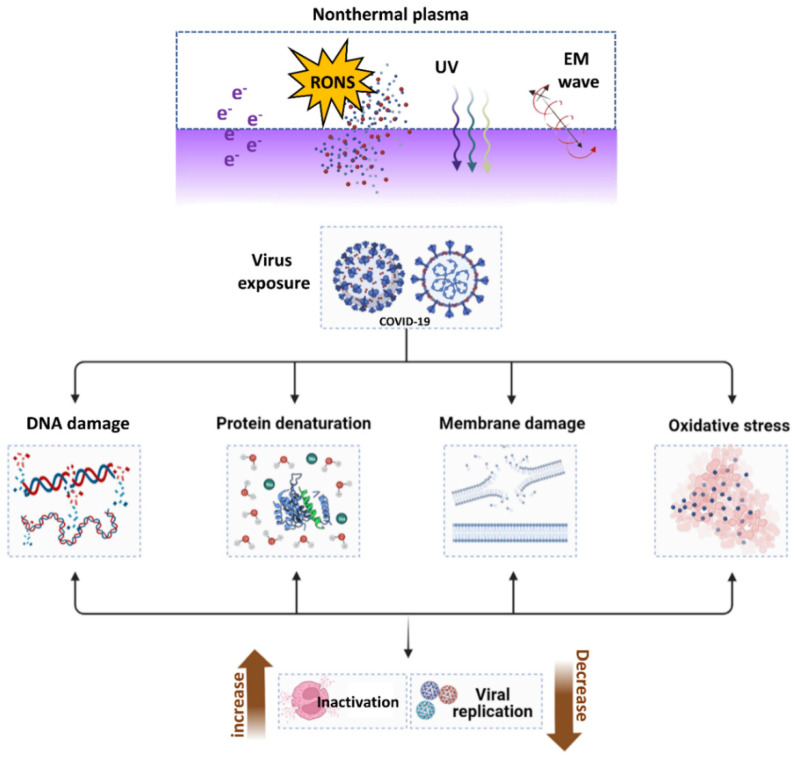
The mechanisms of NTAP in microorganisms in response to biological challenges. The NTAP is a cocktail of reactive species, UV, and RONS that is beneficial in iniactivating COVID-19 through DNA damage, protein denaturation, membrane damage, and oxidative stress, which ultimately results in the virus being rendered inactive or restricted to replication.

Plasma has also been demonstrated to degrade pure SARS-CoV-2 RNA and change exterior components of the virus required for attachment, such as the spike protein [114,120,121]. As demonstrated in Figure 10, NTAP exposure to the virus causes DNA and RNA damage, protein denaturation, membrane damage, and oxidative stress, all of which contribute to increased viral inactivation and decreased viral replications. Additionally, vaccination against many pathogenic viruses has always been the most effective antiviral method [122]. During normal virus replication, the spike proteins of the COVID-19 virus bind to the host cell’s ACE2 receptor and enter the cell for replication. When the COVID-19 virus was subjected to NTAP, the binding spike proteins were damaged, which restricted the virus from binding to the ACE2 receptor and infecting the host. Furthermore, NTAP exposure causes oxidative stress, DNA and RNA damage, protein denaturation, and virus membrane damage, as shown in Figure 10. Combining all these effects helps to inactivate or reduce viral replications by using NTAP.

## 7. Conclusions

The COVID-19 pandemic has imposed a burden on the globalized world, exposing defects in supply chains for disinfectants, personal protective equipment, and healthcare resources. It is critical to find effective treatments and medications for these viral diseases. Nations all over the world are fighting COVID-19, and this review represented an important step toward next-generation collective prevention systems and summarized and highlighted key aspects of recent breakthroughs in COVID-19 inactivation vaccines, inhibitors, radiations, and innovative NTAP technologies. Scientists are looking for technologies that have the potential to replace surgical masks, providing comprehensive protection during the lengthy vaccine development cycle. NTAP has emerged as a method of viral inactivation that is safe, effective, and efficient, which might be helpful in the present situation. NTAP can be used to inactivate viruses in a variety of matrices while also activating animal and plant viruses. The scientific community wants to assist the medical community in dealing with the ongoing COVID-19 epidemic by developing techniques that use widely available NTAP technologies. We hope that this review will allow readers to examine the progress made in the fight against COVID-19 by using vaccines, inhibitors, radiation, and NTAP. Furthermore, NTAP technology is not dependent on viral strain, and months or years of research are not required to develop specific vaccines for each novel or emerging viral disease. We believe the NTAP is a promising technique for combating present COVID-19 and other viruses in the future.

## Figures and Tables

**Table 1:** The summary vaccines and its types.

Vaccine Platform	Total Vaccine	Vaccine Type
Protein subunit (PS)	55	Maximum spike protein-based and less combined with nanoparticle (4 no)
Viral vector non-replicating (VVnr)	23	Adenovirus, lentiviral and Sendai viral vector-based
DNA	16	DNA plasmid encoding RDB, S and others
Inactivated virus (IV)	22	Inactivated whole virus or CpG1018
RNA	40	mRNA
Viral vector replicating (VVr)	4	Based on the influenza A virus
Virus-like particle (VLP)	6	expressing S, E, M, and N
VVr + antigen-presenting cell (VVr + APC)	2	-
Live attenuated virus (LAV)	2	Codon deoptimized live attenuated virus
VVnr + antigen-presentingcell (VVnr + APC)	1	Mesenchymal stem cell expressing with S and N
Bacterial antigen-spore expression vector (BacAg-SpV)	1	Oral salmonella enteritidis (3934 vac)

## Data Availability

Not applicable.

## Supplementary Materials

The following supporting information can be downloaded at: https://www.mdpi.com/article/10.3390/cimb44110384/s1, Table S1: Human Coronavirus epidemiology and genomic information; Table S2: Candidates’ clinical RNA and DNA vaccines (WHO, 23 February 2022).
