# Supplementary material for: Review of Developments in Combating COVID-19 by Vaccines, Inhibitors, Radiations, and Nonthermal Plasma
Source: Curr Issues Mol Biol. 2022 Nov 15;44(11):5666–90. doi: 10.3390/cimb44110384 (PMC9689378; doi:10.3390/cimb44110384)
Supplement: Supplementary file 1 [file cimb-44-00384-s001.zip › cimb-2010184-supplementary.pdf]

Supplementary information

Review of developments in combating COVID19 by vaccines, inhibitors, radiations, and nonthermal plasma

Ihn Han[1,2,†,*], Sohail Mumtaz[2,3,†], Dharmendra Kumar Yadav[4], Sekar Ashok Kumar[2], and Eun Ha Choi[1,2,3,*]

Table S1. Human Coronavirus epidemiology and genomic information

Human Coronavirus (HCoV)	Reservoir	Host	Identified country & year	Causes	Incubation period (Days)	Genome feature			Amino acid length (Receptor binding)	Cellular Receptor	Co receptor	Genome order
						Strand	Range (kb)	G+C content (%)				
**Alpha-coronavirus**												
HCoV-229E	Bats	palm civets	1960	S, F, DC, M & D	2-5	+ RNA	27,317	38	407-547	ANPEP	-	5′-ORF1a-ORF1b-S-ORF4a-ORF4b-E-M-N-polyT-3′
HCoV-NL63	Bats	Bats	Netherland, 2004	F, DC & EI	2-4	+ RNA	27,553	34	476–616	ACE2 receptor	Heparan sulphate	5′-ORF1a-ORF1b-S-ORF3-E-M-N-polyT-3′
Beta-coronaviruses												
HCoV-OC43	Mice	Cattle	1967	CC, S, F DC, ST, D & Pn	2-5		30,738	37	15–302	9-O-acetylsialic acids as receptor	Sialic acid	5′-ORF1a-ORF1b-NS2a-HE-S-ORF5-E-M-N-polyT-3′
SARS-CoV	Bats	civet cat	2003 China	CC, F, DC, SB & Pn	2-11		29,751	41	306–527	ACE2	C-type lectins	5′-ORF1a-ORF1b-S-SP-E-M-SP-ORF8-SP-N-polyT-3′
HCoV-HKU1	Mice	Mice	2005 Hong Kong	CC, S, F, DC, ST, M, D & Pn	2-4		29,926	32	15–302	9-O-acetylsialic acids as receptor	Sialic acids	5′-ORF1a-ORF1b-HE-S-ORF4-E-M-N-polyT-3′
MERS-CoV	Bats	Dromedary	2012	DC, SB,	2-13		30,119	41	367–606	Dipeptidyl	-	5′-ORF1a-

		camels	Saudi Arabia	Pn, & D						peptidase-4 (DPP4) receptor		ORF1b-S-NS4b-E-M-N-polyT-3′
SARS-CoV-2	Bats	Bats	2019 China	F, DC, SB, M, Pn, OFD	3-8		29,903	38	333–527	ACE2	C-type lectins	5′-ORF1a-ORF1b-S-ORF3-SP-E-M-SP-ORF8a-ORF8b-N-polyT-3′

Note: S (Sneezing), F (Fever), DC (Dry Cough), ST (Sore Throat), M (Myalgia), D (Diarrhea), F (Febrile), EI (Eye Inflammation), CC (Common Cold), Pn (Pneumonia), SB (Shortness of Breath), OFD (Organ Failure and even Death). ORF (Open reading frame) 1a and ORF 1b non-structural protein common to all group, S- spike protein, E- Envelop protein, M- Membrane protein, N- Nucleocapsid protein, HE- Hemagglutinin esterase, NS2a- Phosphodiesterase activity, SP- structural protein, ORF8-Beta coronavirus

Table S2. Candidates' clinical RNA and DNA vaccines (WHO, 23rd February 2022)

**Type of vaccine**	**Number of doses**	**Route of administration**	**Schedule (Day)**	**Phase**	**Developers**	
**RNA based Vaccine**						
mRNA 1273	2	IM	0 to 24	4	Moderna + national institute of allergy and infection disease (NIAID)	Gilbert, et al., 2021
BNT162b2 (3LNP-mRNAs)	2	IM	0 to 21	4	Pfizer/BioNTech+Fosun pharma	Thomas, et al., 2021
mRNA-1273.351, lipid nanoparticle (LNP)-encapsulated mRNA	3	IM	0-28/56	4	Moderna+ national institute of allergy and infection disease (NIAID)	
mRT555	2	IM	0-21	4	Sanofi Pasteur and Translate Bio	

CVnCoV	2	IM	0 to 21	3	Cure Vac AG	
SARS-CoV-2 mRNA vaccine (ARCOV)	2	IM	0-14/28	3	Academy of military science (AMS) Walvax biotechnology and Suzhou Abogan and bioscience	
ARCT-154 mRNA	2	IM	0-28	3	Arcturus Therapeutic, Inc.	
Ds-5670a	2	IM	NR	2/3	Daiichi Sankyo Co Ltd.	
mRNA-1273.211; booster candidate complaining mRNA-1273+mRNA-1273.351	1	IM	0	2/3	ModernaTx, Inc.	
ARCT-021	NR	IM	NR	2	Arcturus Therapeutics	
PTX-COVID19-B	2	IM	0-28	2	Providence Therapeutics	
LNP-nCoVsaRNA	2	IM	NR	1	Imperial college London	
ChulaCoV19 mRNA	2	IM	0-21	1	Chulalongkorn University	
CoV2 SAM (LNP). A self- amplifying mRNA (SAM) lipid nanoparticle (LNP) platform + spike antigen	2	IM	0-30	1	GlaxoSmithKline	
HDT-301-self replicating mRNA vaccine formulated lipid nanoparticle	2	IM	0-28	1	SENAI CIMATEC	
mRNA-1283; mRNA-1283.211	2	IM	0-28	1	ModernaTX, Inc	
mRNA COVID-19	2	IM	TBT	1	Shanghai East Hospital and Stemirna Therapeutics	
LNP-nCOV saRNA-02; saRNA encapsulated lipid nanoparticle	2	IM	0-28	1	MRC/UVRI and Uganda research unit	
HDT-301	1-2	IM	0-56	1	HDT Bio	
VLPCOV-01,	2	IM	NR	1	VLP Therapeutic Japan GK	
EXG-5003, RBD SARS-CoV-2-spike protein	1	ID	0	1/2	Elixirgen Therapeutic, Inc	
ARCT-165 mRNA	2	IM	0-29	1/2	Arcturus Therapeutic, Inc.	
ARCT-021 mRNA	2	IM	0-29	1/2	Arcturus Therapeutic, Inc.	
EG-COVID	3	IM	0-21-42	1/2	EyeGene Inc.	

**DNA based vaccine**						
INO-4800+electroporation	2	ID	0 + 28	3	Inovio Pharmaceuticals + International Vaccine Institute + Advaccine	
nCov vaccine	3	ID	0 + 28 + 56	3	Zydus Cadila	
AG0301-COVID19	2	IM	0 + 14	2/3	AnGes + Takara Bio + Osaka University	
GX-19N	2	IM	0 + 28	2/3	Genexine Consortium	
Covigenix VAX-001 - DNA vaccines + proteo-lipid vehicle (PLV) formulation	2	IM	0 + 14	1	Entos Pharmaceuticals Inc.	
CORVax12 - Spike (S) Protein Plasmid DNA Vaccine	2	ID	0 + 14	1	OncoSec Immunotherapies; Providence Health & Services	
bacTRL-Spike oral DNA vaccine	1	Oral	0	1	Symvivo Corporation	
Plasmid DNA vaccine SCOV1 + SCOV2. COVIDITY	2	ID/IM	0 + 28	1	Scancell Ltd	
COVIGEN	2	ID or IM	0 + 28	1	University of Sydney, Bionet Co., Ltd<br>Technovalia	
SARS-CoV-2 DNA vaccine (delivered IM followed by electroporation)	2	IM	0 + 21	1	The University of Hong Kong; Immuno Cure 3 Limited	
Prophylactic pDNA Vaccine Candidate Against COVID-19	3	IM	0 + 21 + 42	1	Imam Abdulrahman Bin Faisal University	
GLS-5310	2	ID	0 + 56	1/2	GeneOne Life Science, Inc.	
COVID-eVax, a candidate plasmid DNA vaccine of the Spike protein	2	IM	0 + 28	1/2	Takis + Rottapharm Biotech	
AG0302-COVID19	2-3	IM	0 + 14 + 28	1/2	AnGes, Inc/Osaka University	
VB10.2129, a DNA plasmid vaccine, encoding the receptor binding domain (RBD)	1-2	IM	0 + 21	1/2	Vaccibody AS	

VB10.2210, DNA plasmid vaccine, encodes multiple immunogenic and conserved T cell epitopes spanning multiple antigens across the SARS-CoV-2 genome.	1-2	IM	0 + 21	1/2	Vaccibody AS	
